# Glycosyl-Phosphatidyl-Inositol (GPI)-Anchors and Metalloproteases: Their Roles in the Regulation of Exosome Composition and NKG2D-Mediated Immune Recognition

**DOI:** 10.3389/fcell.2016.00097

**Published:** 2016-09-12

**Authors:** Sheila López-Cobo, Carmen Campos-Silva, Mar Valés-Gómez

**Affiliations:** Department of Immunology and Oncology, Spanish National Centre for BiotechnologyMadrid, Spain

**Keywords:** GPI-anchored proteins, metalloproteases, DRMs, MICA/B, ULBP, shedding, exosomes, immune evasion

## Abstract

Communication within the immune system depends on the release of factors that can travel and transmit information at points distant from the cell that produced them. In general, immune cells use two key strategies that can occur either at the plasma membrane or in intracellular compartments to produce such factors, vesicle release and proteolytic cleavage. Release of soluble factors in exosomes, a subset of vesicles that originate from intracellular compartments, depends generally on biochemical and lipid environment features. This physical environment allows proteins to be recruited to membrane microdomains that will be later endocytosed and further released to the extracellular milieu. Cholesterol and sphingolipid rich domains (also known as lipid rafts or detergent-resistant membranes, DRMs) often contribute to exosomes and these membrane regions are rich in proteins modified with Glycosyl-Phosphatidyl-Inositol (GPI) and lipids. For this reason, many palmitoylated and GPI-anchored proteins are preferentially recruited to exosomes. In this review, we analyse the biochemical features involved in the release of NKG2D-ligands as an example of functionally related gene families encoding both transmembrane and GPI-anchored proteins that can be released either by proteolysis or in exosomes, and modulate the intensity of the immune response. The immune receptor NKG2D is present in all human Natural Killer and T cells and plays an important role in the first barrier of defense against tumor and infection. However, tumor cells can evade the immune system by releasing NKG2D-ligands to induce down-regulation of the receptor. Some NKG2D-ligands can be recruited to exosomes and potently modulate receptor expression and immune function, while others are more susceptible to metalloprotease cleavage and are shed as soluble molecules. Strikingly, metalloprotease inhibition is sufficient to drive the accumulation in exosomes of ligands otherwise released by metalloprotease cleavage. In consequence, NKG2D-ligands appear as different entities in different cells, depending on cellular metabolism and biochemical structure, which mediate different intensities of immune modulation. We discuss whether similar mechanisms, depending on an interplay between metalloprotease cleavage and exosome release, could be a more general feature regulating the composition of exosomes released from human cells.

Interactions within the immune system frequently depend on the release of soluble factors. In general, two main mechanisms underlie the release of these immune mediators: shedding, generally mediated by enzymatic cleavage, often by members of the metalloprotease (MP) families, and recruitment into vesicles. Extracellular vesicles, often referred to as exosomes, are generated by release of intraluminal vesicles formed in the endocytic pathway while other types of nanoparticles can originate from different cellular compartments (plasma membrane-derived, endocytic nanovesicles, etc). Here, we will discuss the regulation and interplay of these two important mechanisms for release of a range of immune factors, including IL6R, ICAM1, TNFR1, with a particular focus on the ligands for the activating immune receptor NKG2D. Although many questions still remain open, it is interesting to observe that cells in different metabolic or stress-related situations seem to preferentially use only one of these possible mechanisms to release specific proteins. This suggests that studying the interactions that regulate these release mechanisms could advance our understanding of the outcome of the multiple pathological situations in which the secretion of a particular soluble factor plays an important immunomodulatory role.

One critical feature of the biology of extracellular vesicles is that they have a very particular lipid, protein, and nucleic acid composition (Colombo et al., 2014) and the membrane properties of exosomes differ markedly from those of plasma membrane-derived vesicles. This led to the idea that recruitment of cargo to exosomes could depend on both biochemical features intrinsic to these proteins as well as an appropriate lipid environment. Given that a large number of cellular routes are likely involved in the synthesis of the different species of lipids and post-translational modifications that drive selective recruitment of proteins into exosomes, metabolic changes, or pathological situations, like pathogen infection and tumor transformation, that affect these cellular pathways could lead to differential recruitment of cargo to vesicles and, in consequence, to potentially different functional outcomes.

Metalloprotease-mediated cleavage of membrane proteins has been described in many inflammatory and immunological processes. Metalloproteases are expressed in all cell types and their activity can be regulated at various levels, including their recruitment to membrane microdomains and their release in exosomes. In this context, the lipid environment of certain membrane regions might be also involved in the regulation of metalloprotease cleavage. In this review, to explore the interrelationship between exosome recruitment and metalloprotease cleavage, we will discuss, as a model, the regulation of the release of NKG2D-ligands, proteins whose expression is induced by stress and signals for immune activation, but that can also act as decoys reducing the intensity of the immune response when they are secreted from tumor cells. We will first discuss some post-translational modifications that preferentially direct proteins, including NKG2D-Ligands, to exosomes; we will revisit some aspects affecting the biology of NKG2D-L, in particular, their release to extracellular milieu and how the process of NKG2D-L vesicle enrichment is further regulated by metalloproteases. We will end by introducing other examples of proteins that also can be released either as part of vesicles or as enzymatically cleaved species.

## Protein recruitment to exosomes: post-translational modifications and glycosyl-phosphatidyl-inositol (GPI) anchors

The protein composition of exosomes has been studied in depth in the last few years and there are now several databases with information obtained from proteomics analysis of exosomes isolated from different cell lines and human materials (Kalra et al., 2012; Mathivanan et al., 2012). Apart from the general enrichment for tetraspanins and endosomal proteins, exosomes also favorably include proteins that have undergone modifications with lipids such as fatty acylation (Shen et al., 2011). Additionally, many glycosyl-phosphatidyl-inositol (GPI) anchored proteins are also found in exosomes, including the complement regulator proteins CD55 and CD59 (Clayton et al., 2003) or the cellular prion protein (PrP^C^) (Fevrier et al., 2004). The lipid environment by itself probably accounts for the recruitment of GPI-anchored proteins (GPI-AP) to exosomes, since the GPI anchor is preferentially embedded in detergent resistant membrane regions (DRMs, membrane microdomains also known as lipid rafts) through the fatty acids that compose the phospholipidic phosphatidyl inositol (PI) moiety. For example CD73, Gce1, are GPI proteins that are found in DRMs, and this is related to their sorting on exosomes (Muller et al., 2009). Also, the GPI-anchor of the prion protein is associated with saturated raft lipids (Taylor and Hooper, 2006).

**Figure 1:**
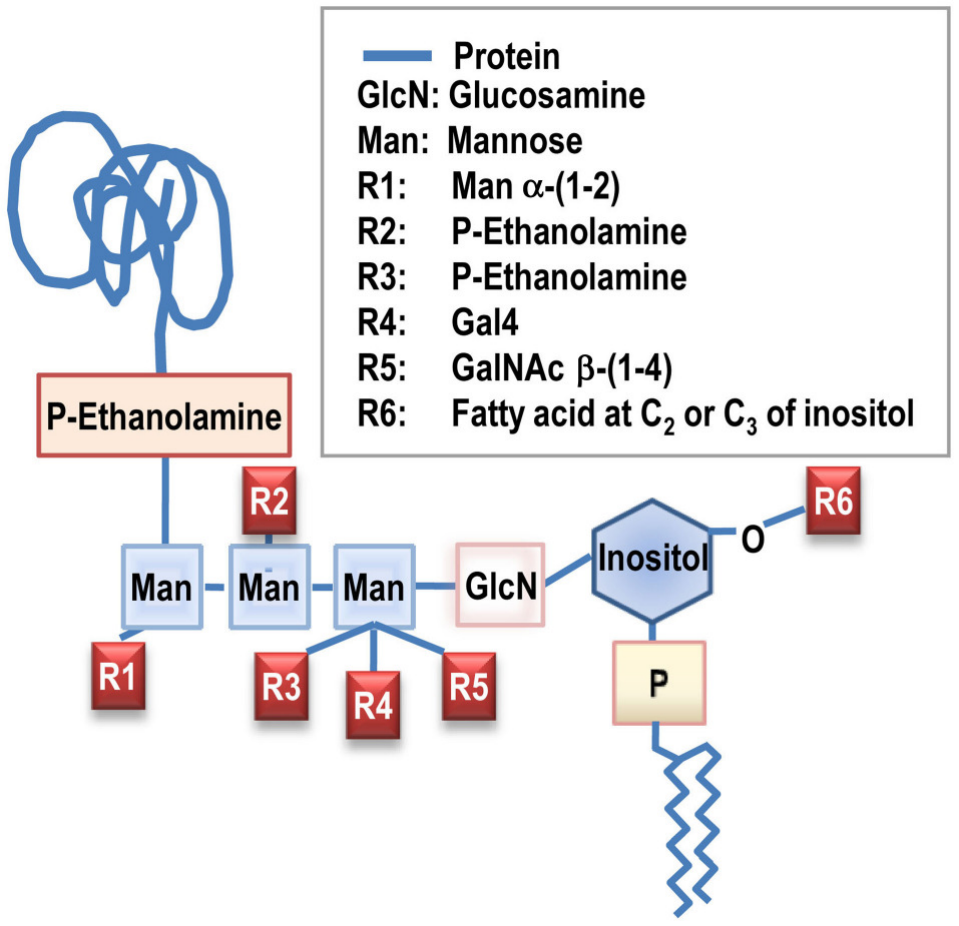
**GPI anchor structure**. Glycosyl-phosphatidyl-inositol (GPI) anchors are glycolipid moieties that allow membrane attachment for a large number of proteins.

The GPI anchor is a glycolipid structure pre-formed at the Endoplasmic Reticulum (ER) before being transferred to the nascent GPI-AP (For review, Paladino et al., 2015). It has a conserved core of PI, linked to a glycan backbone consisting of a single glucosamine residue and three mannoses. These sugars are linked to an ethanolamine phosphate that is attached to the protein via an amide bond (Figure 1). This core can have different lateral modifications that consist of the attachment of other glycans and/or ethanolamine molecules. In humans, there exist many GPI-AP with very distinct functions, and there are many lines of evidence to indicate that GPI anchors are more than just a membrane attachment for proteins, however, their trafficking and biology is only starting to be understood. Generation of GPI-AP requires the involvement of several multi-enzymatic complexes that participate in the synthesis and the transfer of the GPI moiety to the amino acid backbone of the protein (Kinoshita et al., 2013; Kinoshita, 2014). It is worth emphasizing here that the newly synthesized GPI-AP initially interact with the ER membrane through a short transmembrane (TM) and short cytoplasmic region before being transferred to a pre-made GPI anchor and that the efficiency of this process might also affect the fate of the protein. Notably, once the protein is bound to the GPI, it does not have any cytoplasmic moiety, which stresses the importance of the lipid environment for their trafficking and the need for bridging with other TM proteins to allow GPI-AP to interact with the intracellular milieu. This is of particular importance since GPI-AP have a high oligomerization rate.

## Lipids, detergent-resistant membrane microdomains, and exosomes

Perhaps because the study of lipids is complex and difficult, there are fewer reports describing the lipid composition of exosomes than those on exosomal proteomics analyses. Nevertheless, several studies have described the particular enrichment of sphingomyelin (SM), phosphatidyl-serine (PS), cholesterol (Ch), and saturated fatty acids in exosomes purified from different cell types, while ceramide and Lyso-bis-phosphatidic acid (LBPA) content seemed to vary in the different studies (Laulagnier et al., 2004, 2005; Llorente et al., 2013; Osteikoetxea et al., 2015a). This particular composition probably accounts for the noteworthy effect of detergents on exosomal membranes that also distinguishes them from plasma membrane-derived vesicles: exosomes are quite sturdy and can resist treatment with detergents that would solubilize regular membranes (Wubbolts et al., 2003; Osteikoetxea et al., 2015b). Similarly, the rigidity of exosome membranes has been described to be higher than that of the plasma membrane of their originating cell (Laulagnier et al., 2004). Indeed, the composition and rigidity of exosomal membranes is reminiscent of the regions of plasma membrane known as lipid rafts or detergent resistant membranes (DRMs; Ikonen, 2001). So, it was rapidly proposed that exosomes would contain rafts (de Gassart et al., 2003) and, in fact, the membrane regions containing DRMs can be endocytosed to form intraluminal vesicles that are then released as exosomes (Tan et al., 2013). DRMs are regions of the plasma membrane rich in Ch and sphingolipids that function as signaling platforms and can play important roles in cell trafficking (Staubach and Hanisch, 2011). DRMs have been described to be important in protein sorting, endocytosis, and viral and bacterial signaling. Although they share many proteins and features, there are still some differences between exosomes and DRMs (Dubois et al., 2015). For example, caveolin and flotillins are lipid raft scaffolding proteins that not always appear in exosomes. Moreover, the composition of lipid rafts and exosomes from a given cell can vary due to tumor transformation or other metabolic changes.

Lipid rafts are rich in GPI-AP, certain transmembrane and acylated proteins, another shared feature with exosomes (Simons and Ikonen, 1997). The enrichment of GPI-AP in DRMs occurs in their trafficking route from Golgi toward the apical membrane in polarized cell and this is consistent with the fact that disruption of DRM formation affects the apical trafficking of GPI-AP (Zurzolo and Simons, 2016). The link between DRMs and GPI-AP is of particular importance in the development of transmissible spongiform encephalopathies, since the conversion of the cellular prion protein PrP^C^ to the disease causing protein PrP^Sc^, occurs when the protein is targeted to the membrane microdomains (Taylor and Hooper, 2006). Interestingly, PrP^C^ regulation also involves cleavage by metalloproteases of the ADAM family (Taylor et al., 2009) and can be released in exosomes (Fevrier et al., 2004), although how these mechanisms could be related is not clear in this context.

## NKG2D-Ligands: an example of differential secretion with functional consequences in the immune response

NKG2D is one of the best characterized activating receptors in human NK cells (Bauer et al., 1999), that also acts as a co-stimulating receptor in CD8^+^ αβ^+^ T cells and γδ^+^ T cells (Das et al., 2001; Groh et al., 2001). In human, it is constitutively expressed in all these populations of effector cells. Upon interaction with its ligands at the cell surface of target cells, NKG2D originates a transduction signal cascade, through the adaptor molecule DAP10 (Upshaw et al., 2006), leading to activation of the perforin-mediated cytolytic response and elimination of the target cell (Hayakawa et al., 2002). However, when the NKG2D receptor is occupied by soluble NKG2D-L the effector cell becomes unresponsive, presumably because the receptor is blocked and/or internalized (Groh et al., 2002).

The study of the ligands for the immune activating receptor NKG2D led to the identification of a large number of different proteins that belong to two genetic families. As we will describe below, certain NKG2D-Ligands are TM proteins and other GPI-AP. Also, some of them are cleaved by metalloproteases, while other are released in exosomes; some NKG2D-Ligands associate with DRM, while others are outside these microdomains, and all of them are able to bind the same receptor with important functional consequences in health and disease. Thus, many cellular pathways can be involved in the biology of the interaction of this receptor with its ligands. The regulation of cellular processes like exosome release and metalloprotease cleavage becomes, in fact, a very important issue for the NKG2D-mediated activation as well as for the evasion of tumors and pathogens from this response. In consequence, the characterization of the fate of these proteins in different situations, in health and stress, is of great significance for modulation of the immune response.

**Figure 2:**
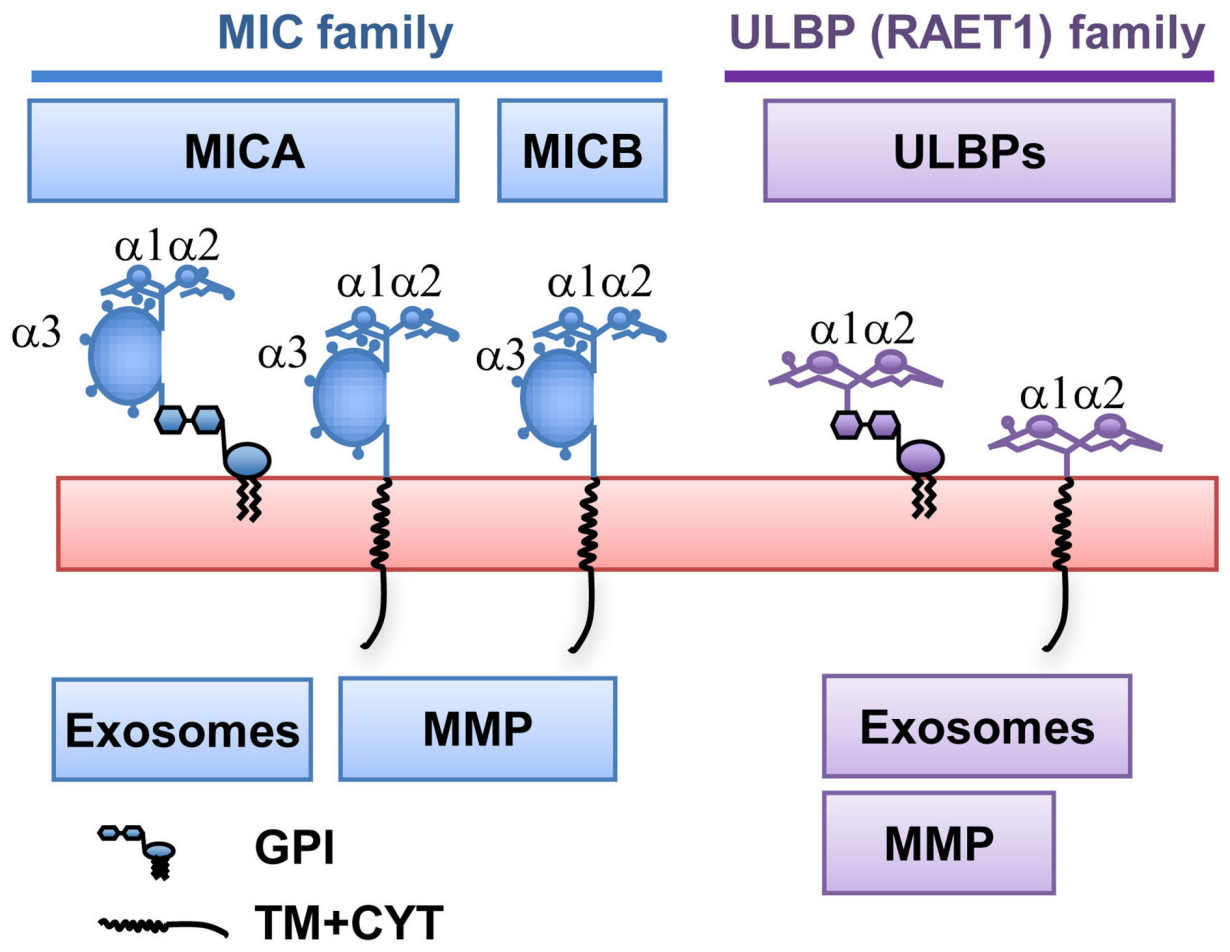
**NKG2D-Ligand families**. NKG2D-ligands belong to two families of MHC class I-related proteins, MICA/B and ULBPs (also known as RAET1). Members with either transmembrane (TM) and cytoplasmic (CYT) domains or Glycosyl-Phosphatidyl-Inositol (GPI)-anchored exist in both families. They are released to the supernatant either as part of exosomes or after matrix metalloprotease (MMP) cleavage.

NKG2D-ligands are MHC-related proteins induced by cellular stress that can be classified in two genetic families: the MIC family (Major Histocompatibility Complex Class-I related Chain) and the ULBP family (UL16-binding proteins, also known as RAET1-A-E; Bahram et al., 1994; Bauer et al., 1999; Cosman et al., 2001). Like conventional MHC class I molecules, MICA/B proteins contain α1, α2, and α3 domains, however, they do not associate with β2-microglobulin and they do not present antigenic peptides (Groh et al., 1996; Figure 2). MICA and MICB are very polymorphic genes and more than 70 MICA and 30 MICB alleles have been described (Steven GE Marsh, Anthony Nolan Research Institute, http://hla.alleles.org/terms.html). Polymorphic residues are found throughout MICA and MICB molecules, however, an interesting polymorphism occurs at the transmembrane region that leads to different biochemical features and affects the trafficking of the molecule (Table 1). The TM region of MICA alleles can differ because of nucleotide insertions (in many of them GCT triplets) leading to the increase in the number of Ala residues in the TM (Mizuki et al., 1997; Ota et al., 1997). In another group of MICA alleles, there is an insertion of a single nucleotide just before the TM region, leading to a frame shift that makes the molecule shorter and acquires the signal for a GPI linkage. A further classification of the MICA alleles, depending on the length of their cytoplasmic and TM regions, is also used and, in this case, all the alleles that contain 4 Ala are called A4, 5 Ala A5, and so on, while the short TM MICA alleles due to a frame shift are called MICA5.1. The existence of either TM or GPI-anchored MICA alleles is important for the biology of these NKG2D-L since, as will be discussed below, they are released by different mechanisms.

**Table 1:**
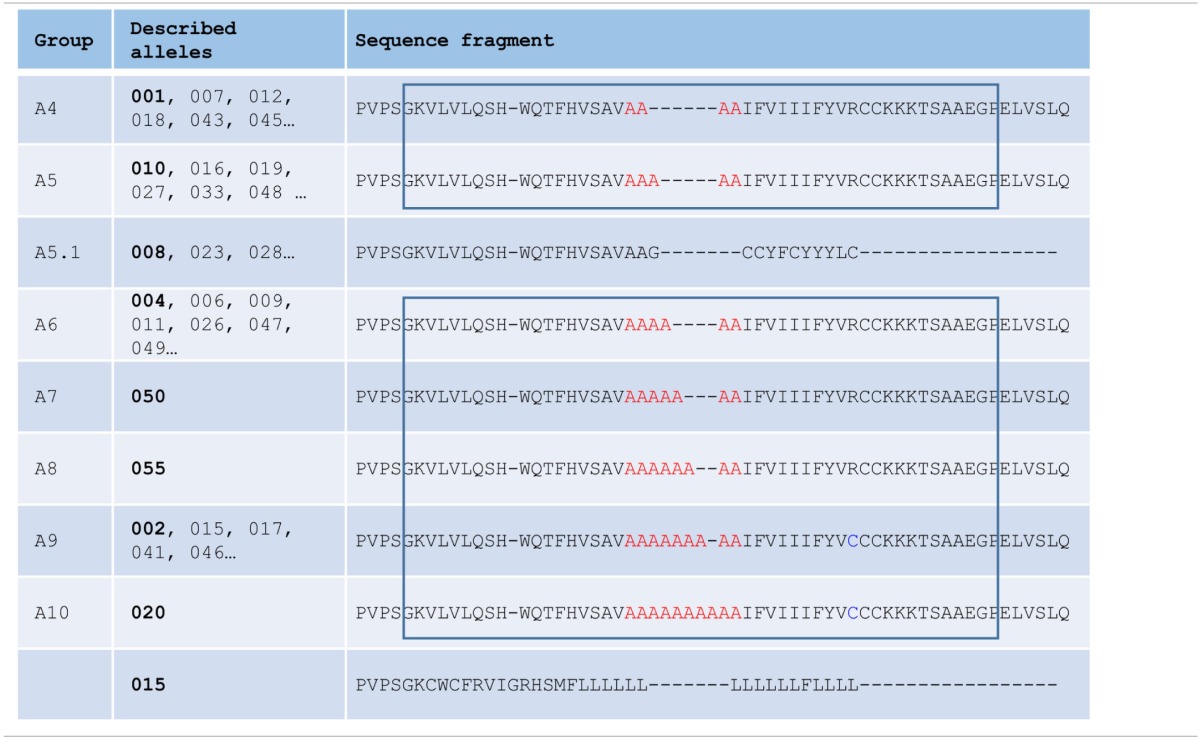
**MICA alleles classification based on the length of the transmembrane region**.

Analysis of the MICA gene revealed a trinucleotide repeat (GCT) polymorphism on exon 5 coding for Alanine. MICA alleles have been classified in groups named A4, A5, A6, A7, A8, A9, and A10, according to the number of Alanine repetitions within the transmembrane region (exon 5, boxed). An additional group called A5.1 consists of an additional nucleotide insertion (GGCT), which in the case of MICA^*^008 has been shown lead to GPI anchoring of the protein. Bold: allele sequence represented. MICA^*^015 does not belong to any group.

Regarding the ULBP family, six different proteins have been described, all of them related to MHC class I, but only containing the α1 and α2 domains and no α3 domain (Cosman et al., 2001; Chalupny et al., 2003; Eagle et al., 2009b; Figure 2). Most MICA alleles, MICB, ULBP4, and ULBP5 are transmembrane proteins (Bacon et al., 2004) whereas ULBP1, ULBP2, ULBP3, ULBP6, and MICA^*^A5.1 alleles (the latter present in around 50% of all the populations studied) are expressed at the cell surface as GPI anchored molecules (Ashiru et al., 2013). ULBP2 and 5 have been shown to be potentially expressed as either GPI or TM proteins (Ohashi et al., 2010; Fernandez-Messina et al., 2011) (For Review, Fernandez-Messina et al., 2012).

The existence of such a large number of ligands with distinct biochemical features for a single receptor may reflect a differential role for different ligands in immune surveillance or an evolutionary response to selective pressures exerted by pathogens.

The expression of a particular NKG2D-L mRNA does not always imply its presence at the cell surface. Instead, the protein can suffer post-transcriptional and post-translational regulation (Heinemann et al., 2012) and modifications that direct it for retention (recycling into endosomal compartments; Aguera-Gonzalez et al., 2009; Eagle et al., 2009a), or extracellular release (Groh et al., 2002; Waldhauer and Steinle, 2006) which occurs in many tumors. These modifications can change the cell fate, and allow a putative target cell to avoid recognition and cytotoxic attack.

The vast majority of healthy cells lack NKG2D-L expression, however, NKG2D-L are found on the surface of cells undergoing different kinds of stress such as tumoral transformation, viral and bacterial infection or autoimmune diseases (For review, Gonzalez et al., 2008). Cytokines also affect the expression of these ligands, for example, while TGFβ or IFN-γ downregulates MICA and ULBP2 (Schwinn et al., 2009; Yadav et al., 2009), IFN-α upregulates its expression (Zhang et al., 2008). Moreover, several viruses encode genes that result in proteins that hijack one or several NKG2D-L, highlighting the importance of this activating receptor in the immune system (Reyburn et al., 2015).

NKG2D-L are expressed in a wide range of cancers, with preferential expression of MICA and MICB in many solid tumors, whereas ULBPs are found in several hematological malignancies, gliomas, and melanomas (Nausch and Cerwenka, 2008; Baragano Raneros et al., 2014b). The detection of particular NKG2D-L in tumors and sera from cancer patients has been associated with prognosis, diagnosis or treatment free-survival in several studies (Spear et al., 2013; Baragano Raneros et al., 2014a). In general, a correlation between more NKG2D-L in serum and poor prognosis has been described (Fernandez-Messina et al., 2012), although the opposite has also been reported (Samuels et al., 2015). A higher expression of these ligands in tumor cells probably mediates an improved immune response, with a better chance for tumor elimination and an improved prognosis. However, the release of NKG2D-L from the malignant cells might represent a strategy to prevent NKG2D-dependent immune response.

**Figure 3:**
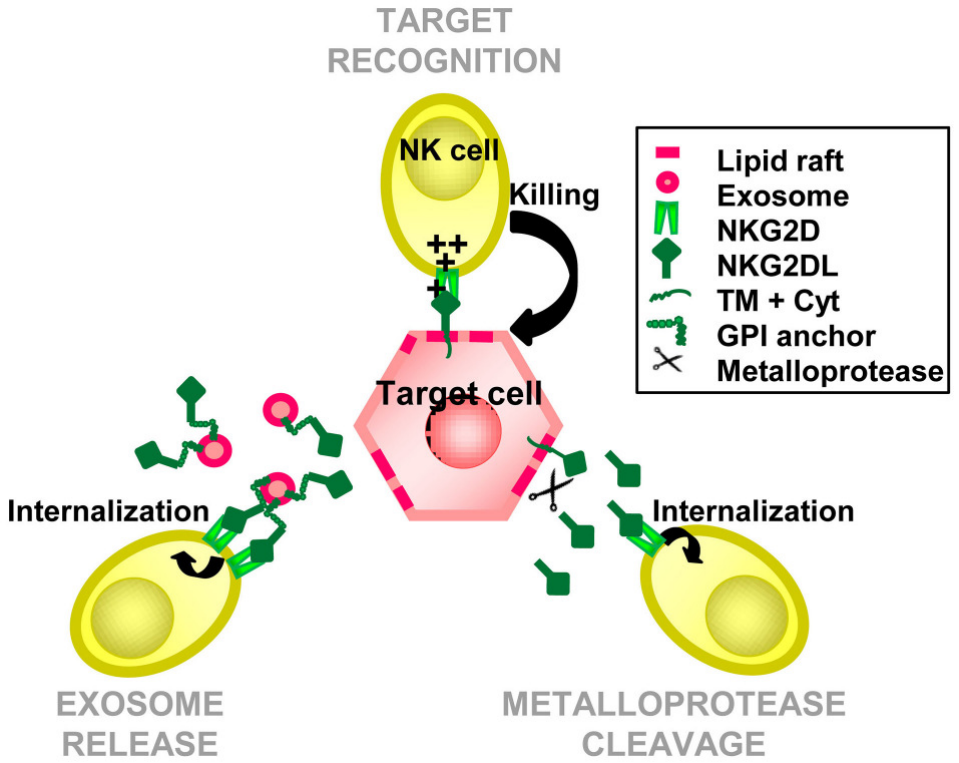
**NKG2D-ligands biology**. NKG2D-ligands (NKG2DL) are recognized by NKG2D on the target cell surface leading to the activation of the degranulation machinery by the effector cell, and target cell death. NKG2DL can be recruited to detergent resistant membranes, also known as lipid rafts, and be either cleaved by metalloproteases, resulting a soluble ligand, or included in exosomes, resulting multimeric membrane-bound ligands. The interaction with soluble NKG2DL, either as cleaved proteins or as part of exosomes, can block and downmodulate the receptor and lead to immune evasion.

The release of NKG2D-L from the cell surface in their soluble form (sNKG2D-L) could have at least three consequences: a reduction in NKG2D-L density at the tumor cell surface, blocking of the NKG2D-binding site and, finally, internalization and downmodulation of the NKG2D receptor on effector cells upon persistent engagement of the sNKG2D-L (Salih et al., 2008; Ashiru et al., 2010). So, knowing the cell biology of NKG2D-L is crucial to understand the regulation of NKG2D-mediated responses and how tumors or viruses evade from this response. However, given the heterogeneity of NKG2D-L, NKG2D modulation can occur in many different ways. In fact, two major mechanisms for the release of NKG2D-L have been described up to now, metalloprotease cleavage and release via exosomes (Figure 3).

## Release of soluble NKG2D ligands: metalloproteases and exosomes

In human, there are three families of Zn-dependent metalloproteases (MPs) each one encoding a large number of genes: matrix metalloproteases (MMPs), the ADAM (a disintegrin and metalloproteinase) family, and ADAM with thrombospondin motifs (ADAM-TS). MPs degrade extracellular matrix and they have an important role in tumor migration. Both MMPs and ADAMs have been suggested to participate in metastasis in multiple cancers and their role as biomarkers for tumor prognosis has been proposed (Noel et al., 2012). Several members of the Zn-dependent MP family have been implicated in the proteolytic shedding of NKG2D-L from tumor cells. MMP9, MMP14, ADAM9, ADAM10, and ADAM17 [also known as Tumor Necrosis Factor (TNF)-a Converting Enzyme (TACE)] have been reported to be involved in MICA and MICB cleavage in different cellular contexts (Salih et al., 2002; Waldhauer and Steinle, 2006; Liu et al., 2010; Sun et al., 2011; Chitadze et al., 2013). ADAM17 and MMP9 have been observed to cleave both MICA and MICB from tumoral cells (Waldhauer et al., 2008; Boutet et al., 2009; Yamanegi et al., 2012) and ADAM15 plays a key role in the regulation of MICB shedding (Duan et al., 2013). GPI-anchored ULBPs can also be cleaved by metalloproteases of the ADAM family (ADAM10 and ADAM17), in transfectants and tumor cells. It is interesting to note that the kinetics for metalloprotease cleavage of different NKG2D-L proteins can be very different and, probably, depends on both susceptibility to the metalloprotease and other cell trafficking properties of each member of the family. In fact, the decrease of both ULBP1 and MICB from the plasma membrane has been shown to depend also on endocytosis of the protein and, in the case of ULBP1, rapid proteasomal degradation (Aguera-Gonzalez et al., 2009; Fernandez-Messina et al., 2016). In consequence, ULBP1 shows a slower kinetics for accumulation in the supernatant than ULBP2 (Fernandez-Messina et al., 2010).

However, the role of metalloproteases regulating the composition of exosome-cargo is not limited to their action at the plasma membrane. It has also been reported that exosomes themselves can be a platform for shedding. Different metalloproteases are found in exosomes, perhaps because at least some are recruited to DRMs, like ADAM17 (Tellier et al., 2006), where they can be responsible for the release of their substrates. The most studied metalloprotease in this context is ADAM10, which is necessary for CD23 incorporation into exosomes (Mathews et al., 2010), but is also related to exosomal CD44 and L1 cleavage and release (Stoeck et al., 2006). Moreover, metalloproteases can themselves be targets for cleavage in exosomes, too, for example, ADAM15 can be shed by the action of serine proteases (Lee et al., 2015), and exosomal MMP14 is involved in the processing of MMP2 in exosomal fractions of cultured corneal fibroblasts (Han et al., 2014). In fact, a body of research on MMPs released in tumor vesicles is being generated. Tumors also secrete large, PM-derived EVs bearing matrix metalloproteinases (Ginestra et al., 1997; Muralidharan-Chari et al., 2009), which could help migration of tumor cells within a solid tissue.

The second main mechanism for release of NKG2D-L is recruitment to exosomes. Several proteins belonging to the two families of NKG2D-L have been detected in exosomes, mainly ULBP1, ULBP3, and MICA^*^008. This MICA allele corresponds to the group of MICA alleles, called A5.1, containing a point mutation leading to GPI membrane attachment. This GPI-anchor has been demonstrated to be responsible for MICA^*^008 recruitment to exosomes (Ashiru et al., 2013). At first glance it could seem that the GPI-anchored NKG2D-L would be mainly released in exosomes while TM NKG2D-L would be cleaved by MP but, given that ULBP2 is also GPI-anchored and mainly MP-cleaved, what determines the mechanism of release used? Interestingly, although several groups of TM MICA alleles, MICB, and ULBP2 are predominantly released by metalloproteases, they can also be released via exosomes in certain cellular contexts. For example, in cells treated with MP inhibitors, ULBP2 can be detected in exosomes (Fernandez-Messina et al., 2010). Several NKG2D-L have also been found in placental exosomes as well as in ovarian cell-derived exosomes or prostate exosomes (Hedlund et al., 2009; Labani-Motlagh et al., 2015). These observations suggest that variations that affect metalloprotease expression, activity, or the possibilities of enzyme and substrate to encounter each other could all modulate the probability of NKG2D-L release as either soluble or exosomal species. For example, efficient proteolytic release of MICA and MICB ligands is promoted by the recruitment of these ligands to DRMs (Boutet et al., 2009), where metalloproteases like ADAM17 reside (Tellier et al., 2006). The recruitment of MICA to these cholesterol-enriched microdomains depends on palmitoylation that has been reported to enhance shedding of this ligand (Aguera-Gonzalez et al., 2011). Thus, alterations in the repertoire or frequency of post-translational modifications are likely to impact shedding of MICA. Moreover, exosomal NKG2D-L seem to affect strongly the intensity of NKG2D-mediated recognition and to downmodulate the receptor more potently than soluble ligands presumably because proteins multimerise on the exosome (Ashiru et al., 2010; Fernandez-Messina et al., 2010; Labani-Motlagh et al., 2015). The recruitment of NKG2D-L to exosomes could be a mechanism adding to the high number of events that facilitate cancer spreading and contribute to the modification of the tumor microenvironment, in particular, the decrease of an effective immune response exerted by tumor exosomes.

Since the NKG2D-L release has important biological consequences, such as blockade and downmodulation of the receptor, decreasing the cytotoxic capacity of the effector cell, several therapeutic strategies have been proposed to disrupt NKG2D-L release with the aim of reinforcing antitumor immunity. One of them is blockade of NKG2D-L shedding by pharmacological inhibitors of MMPs and ADAM proteins (MMPI II, MMPI III, GW280264X, or GI254023X; Waldhauer and Steinle, 2006; Waldhauer et al., 2008). Although MP inhibition can be used to control tumor spreading, this treatment would not affect exosomal release of NKG2D-L, indeed, depending on the alleles of MICA expressed, it could augment the expression of NKG2D-L in exosomes and thus only partially regulate the effects of cell-free ligands on immune responses mediated by the NKG2D receptor. Similarly, chemotherapeutic agents have been shown to increase cell surface NKG2D-L, and downregulate mRNA expression of metalloproteases (MMP2, MMP9, ADAM10, or ADAM9). For example, epirubicin, a drug used to treat hepatocellular carcinoma, has been shown to modulate the expression of ADAM10 in HepG2 cells, leading to a decrease in soluble MICA (Kohga et al., 2009, 2010); gemcitabine increased the expression of NKG2D-L in several carcinoma cells and was used successfully in patients prior to infusion with cytokine-activated killer cells (Morisaki et al., 2011, 2014; Xu et al., 2011). Finally, proteasome inhibitors, epigenetic modifying drugs, such as histone deacetylase inhibitors (such as Valproate) and demethylating reagents, may also modulate the production of sNKG2DL (Armeanu et al., 2005; Skov et al., 2005; Vales-Gomez et al., 2008; Butler et al., 2009; Chavez-Blanco et al., 2011; Yamanegi et al., 2012).

## Consequences of the heterogeneity in NKG2D-L release on the immune response

As mentioned above, NKG2D-L have been detected in cancer patient sera and a considerable body of evidence suggests that the amount of soluble NKG2D-L can be related with prognosis. NKG2D-L release can be induced by viral infection and affect immune recognition, for example HIV (Matusali et al., 2013) and HCMV (Esteso et al., 2014). Many *in vitro* experiments have shown that the presence of NKG2D-L in tissue culture supernatants leads to the down-modulation of the NKG2D receptor (Groh et al., 2002). Interestingly, when NKG2D-L have been found in patient sera, this also correlates with lower expression levels of the NKG2D receptor in the immune effector cells (Wu et al., 2004; Hilpert et al., 2012), suggesting a more immunocompromised state of the immune system in these patients. Two main events could be causing the decrease in the detection of NKG2D: blocking, so that the receptor-specific antibody can no longer bind, or internalization caused by cross-linking. The first possibility is very unlikely to be happening in samples isolated from patients if the molecule was released by metalloprotease cleavage. The interaction between NKG2D and its monomeric ligands has, in general, lower affinity in human than the murine system, ranging around the 1–10 micromolar (Raulet et al., 2013). This means that, unless present in an extremely high concentration, soluble ligands will not be found blocking the receptor. Moreover, they will probably separate during the staining process. Although in most serum or plasma samples the amount of soluble NKG2DL is found in the picomolar range, this does not exclude that a higher concentration could be achieved locally *in vivo*. Thus, the effect of the NKG2D system at the site of ligand expression and release could be more important than in peripheral blood. A very different scenario can be envisioned when talking about exosomal NKG2D-L. The fact that exosomal molecules are on a membrane and, therefore, multimeric, will enhance the avidity for the receptor and might cause a longer lasting interaction than that of monomeric proteins. This effect could be observed *in vitro* when comparing the ability to downmodulate the receptor from metalloprotease cleaved vs. exosomal protein: despite lower amounts of the exosomal NKG2D-L protein, as detected by ELISA, the down-modulation caused by these supernatants was greater than that triggered by soluble molecules (Fernandez-Messina et al., 2010). These observations suggest that identifying not only the presence but also the biochemical form of NKG2D-L in patients can help understanding classifying patients more efficiently. In this context, knowing the MICA genotype of these patients will be important (Vales-Gomez, 2015).

In addition to differences in the identity of ligands and affinity of interaction, signaling through the NKG2D receptor is also different in human and mice (the former associating with DAP10 and the latter with both DAP10 and DAP12), and so, the potential effect of soluble NKG2D-L to modulate the immune response can be very different in the two species. In fact, in mice, a high affinity soluble NKG2D-L can actually activate NKG2D-mediated response, leading to the rejection of the tumor cells that secreted it (Deng et al., 2015). However, it is not clear whether a similar mechanism could happen in humans.

That NKG2D plays an important role in the immune system is highlighted by the mechanisms developed by viruses to control NKG2D-mediated response and HCMV is a clear example of this (Reyburn et al., 2015). However, many questions remain still open on how to modulate this receptor and ligand system to help patients. Murine research shows different impacts on autoimmune diseases of the lack of NKG2D (Guerra et al., 2013) and references therein), thus, more research is needed to understand the best way to potentiate the ability of this receptor to target unhealthy cells or to incorporate blocking agents of the NKG2D-L in the clinic.

## Is there a link between DRMs, metalloproteases, GPI-anchors and exosomes for proteins other than NKG2D-L?

Examples of other proteins with dual secretion mechanisms, exosomes and metalloprotease cleavage, can be found in the immune system and outside, however to which point they can parallel the biology of NKG2D-L is certainly not clear. A similar pattern of regulation could be happening for IL6 receptor (IL6R), which can be found in high amounts in human serum as two different versions of secreted IL6R: a soluble IL-6R, generated by proteolytic release of the ectodomain by metalloproteases of the ADAM family (Lust et al., 1992), and the recently described IL6R released in exosomes (Schumacher et al., 2015). Interestingly, the exosomal IL6R migrates faster than the cellular counterpart, suggesting that a post-translational modification might be responsible for the incorporation of the receptor into exosomes and the faster migration in SDS-PAGE. As expected, the exosomal IL6R isoform is released with slower kinetics than the metalloprotease isoform (Cichy et al., 1997). The observation that IL-6R is present in exosomes poses the question of whether the different forms of IL6R exert different functional activities and this could be of importance in the role of this receptor's agonistic functions for proliferative IL-6 responses.

Another cytokine receptor, TNFR1 is also released in exosome-like vesicles (Hawari et al., 2004) and shed by metalloprotease cleavage (Schall et al., 1990). Strikingly, TNF-α, can also be found in exosomes, where it has been suggested to play a role in the pathogenesis of rheumatoid arthritis (Zhang et al., 2006). Another interesting example, is the case of ICAM1, which although susceptible to proteolytic cleavage by ADAM17 (Tsakadze et al., 2006) is released from senescent cells in exosomes. This is particularly striking since senescent cells have an increased expression of ADAM17, as shown by an increased shedding of soluble TNFR1.

The non-immune system prion protein (PrP) is described to be released in exosomes, but, it can also be released in a faster manner by different mechanisms: Alpha cleavage, resulting in a soluble N1 fragment and a C1, GPI anchored fragment, which can also be found in exosomes; beta cleavage, that occurs closer to the N-terminal region and is the mechanism identified as shedding. Finally, the C-terminal end cleavage, which generates a soluble protein, or a soluble C1 if the protein is already cleaved by alpha cleavage (For review, Altmeppen et al., 2013). Interestingly, Wik and coworkers showed that the use of general metalloprotease or ADAM inhibitors reduced C-terminal end cleavage, but that this led to an increase of C1 fragment delivered in exosomes (Wik et al., 2012). This correlation between inhibition of the protein shedding and increased presence in exosomes, is reminiscent of NKG2DL.

Are these just examples of different metabolic situations controlling the outcome of a biological process? Coming back to NKG2D-L, several groups have described, in different cellular models, the release of certain members of both families (MICA/B and ULBPs) by either exosomes or metalloproteases (and different enzymes have been described to affect the same ligands in different contexts). What initially could seem just a mess, is probably reflecting the differential metabolic situation of the different systems used, in which a trafficking route could be affected or the availability to metalloproteases of the different NKG2D-L could be impaired. This idea might be especially relevant in the placenta. Metalloproteases are needed for the controlled invasion of trophoblasts, however, in pregnancies carried to term the expression of the endogenous MP inhibitors (TIMPs) are higher than in miscarriages (Nissi et al., 2013). Moreover, TNFa release has been related to preeclampsia due to the decrease in TIMPs and increase in TACE (Ma et al., 2011). Given that metalloprotease inhibition led to an increase of exosomal ULBP2, in placenta, recruitment of NKG2D-L to exosomes could be favored by the increase of TIMPs and a decrease in MMP susceptibility. The number and variety of proteins that can be released from the cell surface in exosomes or by enzymatic cleavage, makes it important to understand how these two different mechanisms interact to shape the secretory profile of different proteins in a variety of contexts.

Research on the responsible mechanism of secretion, and its specific functional consequence could be very important to identify new pathways of disease pathogenesis and new therapeutical perspectives. Further, it is tempting to speculate that by unmasking biological pathways connecting the different protein release mechanisms, it would be possible to control the recruitment of proteins to a non-desired environment.

## Author contributions

SL, CC, and MV wrote the manuscript and prepared the figures.

### Conflict of interest statement

The authors declare that the research was conducted in the absence of any commercial or financial relationships that could be construed as a potential conflict of interest.
